# Bis[2-(2-hy­droxy­phen­yl)-1*H*-benzimidazol-3-ium] chloranilate

**DOI:** 10.1107/S2414314621011500

**Published:** 2021-11-04

**Authors:** Hiroyuki Ishida

**Affiliations:** a Department of Chemistry, Faculty of Science, Okayama University, Okayama 700-8530, Japan; University of Aberdeen, Scotland

**Keywords:** crystal structure, chloranilic acid, 2-(2-hy­droxy­phen­yl)-1*H*-benzimidazole, hydrogen bond

## Abstract

In the crystal of the title mol­ecular salt {systematic name: bis­[2-(2-hy­droxy­phen­yl)-1*H*-benzimidazol-3-ium] 2,5-di­chloro-3,6-dioxo­cyclo­hexa-1,4-diene-1,4-diolate}, 2C_13_H_11_N_2_O^+^·C_6_Cl_2_O_4_^2−^, the chloranilate anion is located on an inversion centre, so that the asymmetric unit contains one cation and one half of the chloranilate anion. In the crystal, the cation and the anion are connected by a bifurcated N—H⋯(O,O) hydrogen bond, forming a 2:1 unit. The units are linked into a layer lying parallel to (  01) *via* O—H⋯O and N—H⋯Cl hydrogen bonds. Between the layers, a C—Cl⋯π inter­action is observed.

## Abstract 2

In the crystal of the title organic salt, the cation and the anion are connected *via* bifurcated N—H⋯(O,O) hydrogen bonds, forming a centrosymmetric 2:1 unit of the cation and anion. The units are further linked by O—H⋯O and N—H⋯Cl hydrogen bonds.

## Graphical abstract

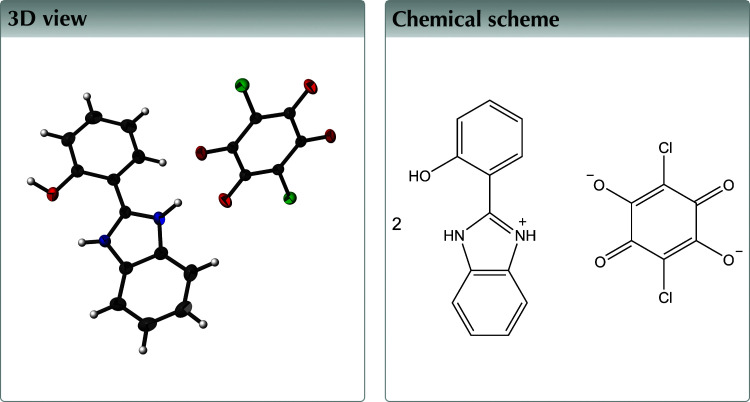

## Structure description

**Figure 1:**
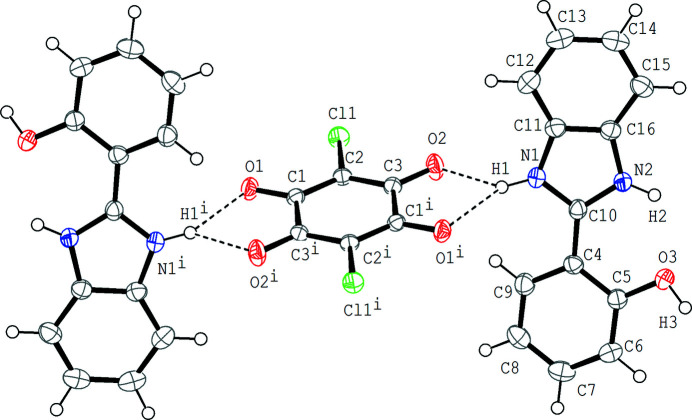
Mol­ecular structure of the title compound, showing the atom-numbering scheme. Displacement ellipsoids are drawn at the 50% probability level and H atoms are shown as small spheres of arbitrary radii. Dashed lines indicate the bifurcated N—H⋯(O,O) hydrogen bonds.

**Figure 2:**
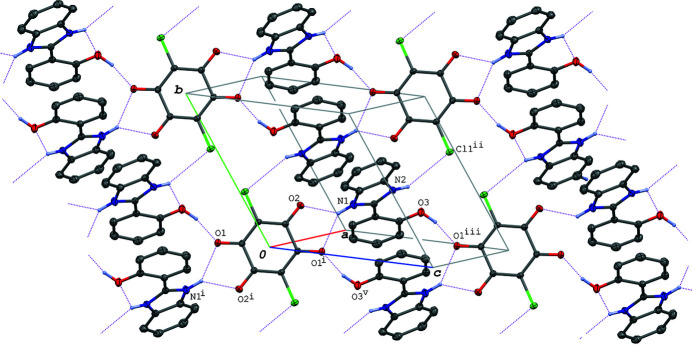
A packing diagram of the title compound, showing the hydrogen-bonded layer structure formed *via* the N—H⋯O, O—H⋯O and N—H⋯Cl hydrogen bonds (magenta dotted lines). H atoms not involved in the hydrogen bonds are omitted for clarity. [Symmetry codes: (i) −*x*, −*y*, −*z*; (ii) −*x* + 1, −*y* + 1, −*z* + 1; (iii) *x* + 1, *y*, *z* + 1; (v) −*x* + 1, −*y*, −*z* + 1.]

We have prepared the title compound in order to continue our studies of *D*—H⋯*A* hydrogen bonding (*D* = N, O or C; *A* = N, O or Cl) in chloranilic acid–organic base systems (Gotoh & Ishida, 2017*a* ▸,*b* ▸, 2018 ▸, and references therein). In the cation, the C4–C9 benzene ring and the C10/N1/C11–C16/N2 benzimidazolium ring system are twisted to each other with a dihedral angle of 17.95 (7)°. An intra­molecular N—H⋯O hydrogen bond (N2—H2⋯O3; Table 1 ▸) is observed. In the crystal, the chloranilate anion is located on an inversion centre, and the cation and the anion are connected by a bifurcated N—H⋯(O,O) hydrogen bond [N1—H1⋯(O1^i^,O2); symmetry code as given in Table 1 ▸], forming a cation–anion 2:1 unit (Fig. 1 ▸). The 2:1 units are further linked into a layer parallel to the ( 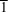 01) plane *via* O—H⋯O and N—H⋯Cl hydrogen bonds (O3—H3⋯O1^iii^ and N2—H2⋯Cl1^ii^; Fig. 2 ▸, Table 1 ▸). A C—Cl⋯π inter­action [C2—Cl1⋯*Cg*3^iv^; Cl1⋯*Cg*3^iv^ = 3.6539 (10) Å and C2—Cl1⋯*Cg*3^iv^ = 139.21 (5)°; symmetry code: (iv) *x*, *y*, *z* − 1] is observed between the layers, where *Cg*3 is the centroid of the C11–C16 ring.

## Synthesis and crystallization

Single crystals of the title salt were obtained by slow evaporation from a methanol solution of chloranilic acid with 2-(2-hy­droxy­phen­yl)-1*H*-benzimidazole in a *ca* 1:1 molar ratio at room temperature [150 ml methanol solution of chloranilic acid (0.45 g) and 2-(2-hy­droxy­phen­yl)-1*H*-benzimidazole (0.45 g)].

## Refinement

Crystal data, data collection and structure refinement details are summarized in Table 2 ▸.

## Supplementary Material

Crystal structure: contains datablock(s) global, I. DOI: 10.1107/S2414314621011500/hb4396sup1.cif

Structure factors: contains datablock(s) I. DOI: 10.1107/S2414314621011500/hb4396Isup2.hkl

Supporting information file. DOI: 10.1107/S2414314621011500/hb4396Isup3.cml

CCDC reference: 2119367

Additional supporting information:  crystallographic information; 3D view; checkCIF report

## Figures and Tables

**Table 1:** Hydrogen-bond geometry (Å, °)

*D*—H⋯*A*	*D*—H	H⋯*A*	*D*⋯*A*	*D*—H⋯*A*
N1—H1⋯O2	0.91 (3)	2.01 (3)	2.7635 (18)	139 (3)
N1—H1⋯O1^i^	0.91 (3)	2.16 (3)	2.9336 (18)	142 (3)
N2—H2⋯O3	0.878 (18)	2.138 (19)	2.6704 (19)	118.4 (15)
N2—H2⋯Cl1^ii^	0.878 (18)	2.823 (18)	3.5907 (14)	146.9 (16)
O3—H3⋯O1^iii^	0.94 (3)	1.73 (3)	2.6524 (18)	165 (3)

Symmetry codes: (i) 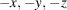 ; (ii) 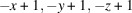 ; (iii) 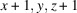 .

**Table 2:** Experimental details

Crystal data	
Chemical formula	2C_13_H_11_N_2_O^+^·C_6_Cl_2_O_4_^2−^
*M*_r_	629.45
Crystal system, space group	Triclinic, *P* 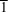
Temperature (K)	180
*a*, *b*, *c* (Å)	8.6694 (11), 9.1751 (13), 10.0313 (14)
α, β, γ (°)	113.654 (4), 95.963 (5), 105.579 (4)
*V* (Å^3^)	683.47 (17)
*Z*	1
Radiation type	Mo *K*α
μ (mm^−1^)	0.29
Crystal size (mm)	0.23 × 0.17 × 0.06

Data collection	
Diffractometer	Rigaku R-AXIS RAPIDII
Absorption correction	Multi-scan (*ABSCOR*; Higashi, 1995 ▸)
*T*_min_, *T*_max_	0.871, 0.983
No. of measured, independent and observed [*I* > 2σ(*I*)] reflections	14095, 3980, 3105
*R*_int_	0.025
(sin θ/λ)_max_ (Å^−1^)	0.704

Refinement	
*R*[*F* ^2^ > 2σ(*F* ^2^)], *wR*(*F* ^2^), *S*	0.038, 0.101, 1.10
No. of reflections	3980
No. of parameters	211
H-atom treatment	H atoms treated by a mixture of independent and constrained refinement
Δρ_max_, Δρ_min_ (e Å^−3^)	0.45, −0.26

Computer programs: *PROCESS-AUTO* (Rigaku, 2006 ▸), *SHELXT2018/2* (Sheldrick, 2015*a* ▸), *SHELXL2018/3* (Sheldrick, 2015*b* ▸), *ORTEP-3 for Windows* (Farrugia, 2012 ▸), *Mercury* (Macrae *et al.*, 2020 ▸), *CrystalStructure* (Rigaku, 2018 ▸) and *PLATON* (Spek, 2020 ▸).

## full crystallographic data

### Crystal data

**Table:**

2C_13_H_11_N_2_O^+^·C_6_Cl_2_O_4_^2^^−^	*Z* = 1
*M**_r_* = 629.45	*F*(000) = 324.00
Triclinic, *P*1	*D*_x_ = 1.529 Mg m^−^^3^
*a* = 8.6694 (11) Å	Mo *K*α radiation, λ = 0.71075 Å
*b* = 9.1751 (13) Å	Cell parameters from 10316 reflections
*c* = 10.0313 (14) Å	θ = 3.4–30.1°
α = 113.654 (4)°	µ = 0.29 mm^−^^1^
β = 95.963 (5)°	*T* = 180 K
γ = 105.579 (4)°	Block, brown
*V* = 683.47 (17) Å^3^	0.23 × 0.17 × 0.06 mm

### Data collection

**Table:**

Rigaku R-AXIS RAPIDII diffractometer	3105 reflections with *I* > 2σ(*I*)
Detector resolution: 10.000 pixels mm^-1^	*R*_int_ = 0.025
ω scans	θ_max_ = 30.0°, θ_min_ = 3.4°
Absorption correction: multi-scan (ABSCOR; Higashi, 1995)	*h* = −12→11
*T*_min_ = 0.871, *T*_max_ = 0.983	*k* = −12→12
14095 measured reflections	*l* = −14→14
3980 independent reflections	

### Refinement

**Table:**

Refinement on *F*^2^	Secondary atom site location: difference Fourier map
*R*[*F*^2^ > 2σ(*F*^2^)] = 0.038	Hydrogen site location: mixed
*wR*(*F*^2^) = 0.101	H atoms treated by a mixture of independent and constrained refinement
*S* = 1.10	*w* = 1/[σ^2^(*F*_o_^2^) + (0.046*P*)^2^ + 0.2103*P*] where *P* = (*F*_o_^2^ + 2*F*_c_^2^)/3
3980 reflections	(Δ/σ)_max_ < 0.001
211 parameters	Δρ_max_ = 0.45 e Å^−^^3^
0 restraints	Δρ_min_ = −0.26 e Å^−^^3^
Primary atom site location: structure-invariant direct methods	

### Special details

**Table:**

Geometry. All esds (except the esd in the dihedral angle between two l.s. planes) are estimated using the full covariance matrix. The cell esds are taken into account individually in the estimation of esds in distances, angles and torsion angles; correlations between esds in cell parameters are only used when they are defined by crystal symmetry. An approximate (isotropic) treatment of cell esds is used for estimating esds involving l.s. planes.
Refinement. Reflections were merged by SHELXL according to the crystal class for the calculation of statistics and refinement. _reflns_Friedel_fraction is defined as the number of unique Friedel pairs measured divided by the number that would be possible theoretically, ignoring centric projections and systematic absences.

### Fractional atomic coordinates and isotropic or equivalent isotropic displacement parameters (Å^2^)

**Table:**

	*x*	*y*	*z*	*U*_iso_*/*U*_eq_	
Cl1	0.09994 (4)	0.34752 (4)	−0.02027 (4)	0.02882 (10)	
O1	−0.14183 (13)	0.01196 (13)	−0.24683 (11)	0.0289 (2)	
O2	0.21238 (14)	0.27933 (13)	0.23324 (12)	0.0346 (3)	
O3	0.75991 (14)	0.23427 (14)	0.69412 (12)	0.0329 (2)	
N1	0.35863 (16)	0.30308 (17)	0.50287 (14)	0.0295 (3)	
N2	0.55002 (15)	0.40290 (15)	0.70561 (13)	0.0254 (2)	
C1	−0.07044 (16)	0.01567 (16)	−0.12934 (14)	0.0209 (3)	
C2	0.04335 (16)	0.15741 (16)	−0.00910 (15)	0.0215 (3)	
C3	0.11500 (16)	0.15402 (16)	0.12180 (15)	0.0220 (3)	
C4	0.59135 (17)	0.19499 (17)	0.47216 (15)	0.0243 (3)	
C5	0.71650 (18)	0.16090 (17)	0.54313 (16)	0.0257 (3)	
C6	0.7897 (2)	0.0516 (2)	0.45539 (18)	0.0322 (3)	
H6	0.873099	0.026322	0.502062	0.039*	
C7	0.7424 (2)	−0.0201 (2)	0.30162 (19)	0.0358 (3)	
H7	0.792239	−0.095973	0.242994	0.043*	
C8	0.6224 (2)	0.0171 (2)	0.23097 (18)	0.0361 (3)	
H8	0.592107	−0.031076	0.124678	0.043*	
C9	0.54818 (19)	0.12356 (19)	0.31562 (17)	0.0305 (3)	
H9	0.466344	0.149222	0.267406	0.037*	
C10	0.50320 (17)	0.29865 (17)	0.55883 (15)	0.0240 (3)	
C11	0.31061 (19)	0.41400 (18)	0.61747 (16)	0.0283 (3)	
C12	0.1694 (2)	0.4589 (2)	0.6176 (2)	0.0384 (4)	
H12	0.084527	0.412181	0.529115	0.046*	
C13	0.1595 (2)	0.5750 (2)	0.7532 (2)	0.0386 (4)	
H13	0.065121	0.609007	0.758876	0.046*	
C14	0.2855 (2)	0.6435 (2)	0.88216 (19)	0.0362 (4)	
H14	0.275017	0.724559	0.973058	0.043*	
C15	0.4247 (2)	0.59789 (19)	0.88273 (18)	0.0330 (3)	
H15	0.509383	0.644482	0.971298	0.040*	
C16	0.43388 (18)	0.48016 (17)	0.74661 (16)	0.0264 (3)	
H1	0.299 (3)	0.241 (3)	0.406 (3)	0.076 (8)*	
H2	0.644 (2)	0.422 (2)	0.763 (2)	0.041 (5)*	
H3	0.812 (3)	0.170 (3)	0.723 (3)	0.065 (7)*	

### Atomic displacement parameters (Å^2^)

**Table:**

	*U*^11^	*U*^22^	*U*^33^	*U*^12^	*U*^13^	*U*^23^
Cl1	0.0365 (2)	0.01955 (15)	0.03055 (19)	0.00738 (13)	0.00309 (14)	0.01391 (13)
O1	0.0337 (6)	0.0260 (5)	0.0248 (5)	0.0086 (4)	−0.0031 (4)	0.0128 (4)
O2	0.0404 (6)	0.0222 (5)	0.0279 (6)	0.0001 (4)	−0.0093 (4)	0.0094 (4)
O3	0.0398 (6)	0.0352 (6)	0.0259 (5)	0.0201 (5)	0.0016 (4)	0.0128 (5)
N1	0.0285 (6)	0.0326 (6)	0.0233 (6)	0.0135 (5)	−0.0001 (5)	0.0083 (5)
N2	0.0262 (6)	0.0227 (5)	0.0233 (6)	0.0085 (5)	0.0008 (5)	0.0078 (5)
C1	0.0220 (6)	0.0210 (6)	0.0222 (6)	0.0094 (5)	0.0053 (5)	0.0106 (5)
C2	0.0252 (6)	0.0166 (6)	0.0238 (6)	0.0073 (5)	0.0047 (5)	0.0103 (5)
C3	0.0225 (6)	0.0191 (6)	0.0228 (6)	0.0066 (5)	0.0034 (5)	0.0088 (5)
C4	0.0267 (7)	0.0201 (6)	0.0255 (7)	0.0072 (5)	0.0057 (5)	0.0101 (5)
C5	0.0278 (7)	0.0233 (6)	0.0261 (7)	0.0076 (5)	0.0050 (5)	0.0123 (5)
C6	0.0327 (8)	0.0328 (8)	0.0378 (8)	0.0170 (6)	0.0107 (6)	0.0179 (7)
C7	0.0404 (9)	0.0329 (8)	0.0387 (9)	0.0167 (7)	0.0202 (7)	0.0151 (7)
C8	0.0443 (9)	0.0342 (8)	0.0264 (8)	0.0119 (7)	0.0110 (7)	0.0106 (6)
C9	0.0322 (8)	0.0313 (7)	0.0273 (7)	0.0102 (6)	0.0039 (6)	0.0137 (6)
C10	0.0256 (7)	0.0215 (6)	0.0238 (7)	0.0063 (5)	0.0019 (5)	0.0112 (5)
C11	0.0321 (8)	0.0264 (7)	0.0286 (7)	0.0132 (6)	0.0073 (6)	0.0123 (6)
C12	0.0353 (8)	0.0438 (9)	0.0406 (9)	0.0198 (7)	0.0058 (7)	0.0199 (8)
C13	0.0389 (9)	0.0401 (9)	0.0498 (10)	0.0230 (7)	0.0190 (8)	0.0243 (8)
C14	0.0462 (9)	0.0297 (7)	0.0392 (9)	0.0176 (7)	0.0202 (7)	0.0163 (7)
C15	0.0407 (9)	0.0257 (7)	0.0302 (8)	0.0110 (6)	0.0090 (6)	0.0105 (6)
C16	0.0284 (7)	0.0225 (6)	0.0289 (7)	0.0085 (5)	0.0064 (5)	0.0122 (6)

### Geometric parameters (Å, º)

**Table:**

Cl1—C2	1.7333 (13)	C6—C7	1.375 (2)
O1—C1	1.2562 (15)	C6—H6	0.9500
O2—C3	1.2392 (16)	C7—C8	1.390 (2)
O3—C5	1.3477 (18)	C7—H7	0.9500
O3—H3	0.95 (2)	C8—C9	1.370 (2)
N1—C10	1.3368 (18)	C8—H8	0.9500
N1—C11	1.3851 (19)	C9—H9	0.9500
N1—H1	0.91 (3)	C11—C16	1.386 (2)
N2—C10	1.3359 (18)	C11—C12	1.393 (2)
N2—C16	1.3860 (19)	C12—C13	1.379 (2)
N2—H2	0.88 (2)	C12—H12	0.9500
C1—C2	1.3872 (18)	C13—C14	1.396 (3)
C1—C3^i^	1.5351 (18)	C13—H13	0.9500
C2—C3	1.4088 (18)	C14—C15	1.379 (2)
C4—C9	1.399 (2)	C14—H14	0.9500
C4—C5	1.4079 (19)	C15—C16	1.387 (2)
C4—C10	1.452 (2)	C15—H15	0.9500
C5—C6	1.390 (2)		
			
C5—O3—H3	107.6 (14)	C9—C8—C7	119.56 (15)
C10—N1—C11	108.93 (12)	C9—C8—H8	120.2
C10—N1—H1	125.9 (17)	C7—C8—H8	120.2
C11—N1—H1	125.2 (17)	C8—C9—C4	120.82 (14)
C10—N2—C16	109.44 (12)	C8—C9—H9	119.6
C10—N2—H2	123.2 (13)	C4—C9—H9	119.6
C16—N2—H2	127.3 (13)	N2—C10—N1	108.68 (13)
O1—C1—C2	125.88 (12)	N2—C10—C4	126.61 (13)
O1—C1—C3^i^	115.72 (11)	N1—C10—C4	124.71 (13)
C2—C1—C3^i^	118.39 (11)	N1—C11—C16	106.88 (13)
C1—C2—C3	123.02 (11)	N1—C11—C12	131.05 (15)
C1—C2—Cl1	118.52 (10)	C16—C11—C12	122.06 (15)
C3—C2—Cl1	118.46 (10)	C13—C12—C11	116.36 (15)
O2—C3—C2	124.66 (12)	C13—C12—H12	121.8
O2—C3—C1^i^	116.80 (11)	C11—C12—H12	121.8
C2—C3—C1^i^	118.54 (11)	C12—C13—C14	121.24 (15)
C9—C4—C5	119.33 (13)	C12—C13—H13	119.4
C9—C4—C10	119.64 (13)	C14—C13—H13	119.4
C5—C4—C10	121.00 (13)	C15—C14—C13	122.52 (15)
O3—C5—C6	122.30 (13)	C15—C14—H14	118.7
O3—C5—C4	118.68 (13)	C13—C14—H14	118.7
C6—C5—C4	119.02 (13)	C14—C15—C16	116.18 (15)
C7—C6—C5	120.51 (14)	C14—C15—H15	121.9
C7—C6—H6	119.7	C16—C15—H15	121.9
C5—C6—H6	119.7	N2—C16—C11	106.04 (12)
C6—C7—C8	120.71 (15)	N2—C16—C15	132.34 (14)
C6—C7—H7	119.6	C11—C16—C15	121.61 (14)
C8—C7—H7	119.6		
			
O1—C1—C2—C3	177.85 (13)	C11—N1—C10—N2	0.01 (17)
C3^i^—C1—C2—C3	−2.5 (2)	C11—N1—C10—C4	179.39 (13)
O1—C1—C2—Cl1	−2.1 (2)	C9—C4—C10—N2	−164.99 (14)
C3^i^—C1—C2—Cl1	177.54 (9)	C5—C4—C10—N2	17.0 (2)
C1—C2—C3—O2	−177.30 (14)	C9—C4—C10—N1	15.7 (2)
Cl1—C2—C3—O2	2.7 (2)	C5—C4—C10—N1	−162.30 (14)
C1—C2—C3—C1^i^	2.5 (2)	C10—N1—C11—C16	0.99 (17)
Cl1—C2—C3—C1^i^	−177.54 (9)	C10—N1—C11—C12	−178.19 (16)
C9—C4—C5—O3	178.22 (13)	N1—C11—C12—C13	179.91 (16)
C10—C4—C5—O3	−3.7 (2)	C16—C11—C12—C13	0.8 (2)
C9—C4—C5—C6	−2.4 (2)	C11—C12—C13—C14	0.4 (3)
C10—C4—C5—C6	175.59 (13)	C12—C13—C14—C15	−1.1 (3)
O3—C5—C6—C7	−179.73 (14)	C13—C14—C15—C16	0.5 (2)
C4—C5—C6—C7	1.0 (2)	C10—N2—C16—C11	1.60 (16)
C5—C6—C7—C8	1.0 (2)	C10—N2—C16—C15	−179.40 (15)
C6—C7—C8—C9	−1.4 (3)	N1—C11—C16—N2	−1.55 (16)
C7—C8—C9—C4	−0.1 (2)	C12—C11—C16—N2	177.72 (14)
C5—C4—C9—C8	2.1 (2)	N1—C11—C16—C15	179.32 (13)
C10—C4—C9—C8	−176.01 (14)	C12—C11—C16—C15	−1.4 (2)
C16—N2—C10—N1	−1.01 (16)	C14—C15—C16—N2	−178.19 (15)
C16—N2—C10—C4	179.62 (13)	C14—C15—C16—C11	0.7 (2)

Symmetry code: (i) −*x*, −*y*, −*z*.

### Hydrogen-bond geometry (Å, º)

**Table:**

*D*—H···*A*	*D*—H	H···*A*	*D*···*A*	*D*—H···*A*
N1—H1···O2	0.91 (3)	2.01 (3)	2.7635 (18)	139 (3)
N1—H1···O1^i^	0.91 (3)	2.16 (3)	2.9336 (18)	142 (3)
N2—H2···O3	0.878 (18)	2.138 (19)	2.6704 (19)	118.4 (15)
N2—H2···Cl1^ii^	0.878 (18)	2.823 (18)	3.5907 (14)	146.9 (16)
O3—H3···O1^iii^	0.94 (3)	1.73 (3)	2.6524 (18)	165 (3)

Symmetry codes: (i) −*x*, −*y*, −*z*; (ii) −*x*+1, −*y*+1, −*z*+1; (iii) *x*+1, *y*, *z*+1.
